# Automation aided optimization of cloning, expression and purification of enzymes of the bacterial sialic acid catabolic and sialylation pathways enzymes for structural studies

**DOI:** 10.1111/1751-7915.13041

**Published:** 2018-01-17

**Authors:** Sneha Bairy, Lakshmi Narayanan Gopalan, Thanuja Gangi Setty, Sathya Srinivasachari, Lavanyaa Manjunath, Jay Prakash Kumar, Sai R Guntupalli, Sucharita Bose, Vinod Nayak, Swagatha Ghosh, Nitish Sathyanarayanan, Rhawnie Caing‐Carlsson, Weixiao Yuan Wahlgren, Rosmarie Friemann, S. Ramaswamy, Muniasamy Neerathilingam

**Affiliations:** ^1^ Centre for Cellular and Molecular Platforms NCBS‐TIFR GKVK Campus Bellary Road Bangalore 560065 Karnataka India; ^2^ Department of Lipid Science CSIR‐Central Food Technology and Research Institute Mysuru 570020 Karnataka India; ^3^ Institute for Stem Cell Biology and Regenerative Medicine GKVK Campus Bellary Road Bangalore 560065 Karnataka India; ^4^ The Institute of TransDisciplinary Health Sciences & Technology (TDU) Bengaluru Karnataka India; ^5^ Manipal Academy of Higher Education Manipal Karnataka India‐576104; ^6^ Department of Chemistry and Molecular Biology University of Gothenburg Box 462 Gothenburg S‐40530 Sweden; ^7^ Centre for Antibiotic Resistance Research (CARe) at University of Gothenburg Box 440 S‐40530 Gothenburg Sweden

## Abstract

The process of obtaining a well‐expressing, soluble and correctly folded constructs can be made easier and quicker by automating the optimization of cloning, expression and purification. While there are many semiautomated pipelines available for cloning, expression and purification, there is hardly any pipeline that involves complete automation. Here, we achieve complete automation of all the steps involved in cloning and *in vivo* expression screening. This is demonstrated using 18 genes involved in sialic acid catabolism and the surface sialylation pathway. Our main objective was to clone these genes into a His‐tagged Gateway vector, followed by their small‐scale expression optimization *in vivo*. The constructs that showed best soluble expression were then selected for purification studies and scaled up for crystallization studies. Our technique allowed us to quickly find conditions for producing significant quantities of soluble proteins in *Escherichia coli*, their large‐scale purification and successful crystallization of a number of these proteins. The method can be implemented in other cases where one needs to screen a large number of constructs, clones and expression vectors for successful recombinant production of functional proteins.

## Introduction

The revolution and the reduction in the cost of DNA sequencing technologies have led to large numbers of protein sequences being determined; however, their structures are being determined at a much lower rate. The rate‐limiting step is in the production of the soluble form of these proteins and subsequent crystallization (Durbin and Feher, 1996; Li and Ismagilov, 2010; Hipolito *et al*., 2014). It is time‐consuming to optimize production of a target protein using different vectors, strains, tags etc., until a well‐expressing, soluble and correctly folded construct can be identified serially (Bashiri *et al*., 2015). Hence, parallel production of recombinant proteins for therapeutic and diagnostic applications is required. Automation of the process has the advantage of reducing errors and providing high sample throughput due to parallel processing of the different steps. The ability to screen many constructs simultaneously in a multiwell format could speed up this screening process considerably.

While there are many semiautomated pipelines available for cloning, expression and purification, there are hardly any pipelines that involve complete automation of these steps; that is individual steps such as PCR set‐up, production, recovery of biomass, purification of DNA and proteins are automated, but certain steps such as transformation step and DNA and protein gel analysis in most pipelines involve some amount of manual intervention. For example, the spreading on LB agar plates is performed by twisting the plates manually after addition of culture solution (Camilo and Polikarpov, 2014). Also, the lid of the agar plate has to be closed manually after drying of the plate is achieved. Another automation platform for cloning, expression and purification is described (Mlynek *et al*., 2014), where some steps such as PCR, plasmid extraction and expression are automated, but would still require manual interventions at multiple points such as gel extraction, transformation and culture inoculation. Similarly, a cell‐free expression‐based method for high‐throughput cloning and expression has been developed (Betton*,* 2004); steps such as protein and DNA analysis are not automated.

Here, we report complete automation of the transformation step and analysis of DNA and proteins. The other steps of cloning, expression and purification have been separately automated using our automation platform. We use a high‐throughput platform to perform cloning, expression and purification of eighteen enzymes involved in sialic acid catabolism and surface sialylation by four different Gram‐negative bacteria (*Haemophilus influenza*,* Fusobacterium nucleatum*,* Pasteurella multocida* and *Vibrio cholera)* (Table S1). Sialic acids are a large family of acidic nine‐carbon sugars (Vimr *et al*., 2004) where the most common type, *N‐*acetylneuraminic acid (Neu5Ac) (Varki, 1992), is found at the terminal positions of glycoconjugates in humans and other deuterostomes (Angata and Varki, 2002). Cell surface sialylation is crucial for a range of biological functions such as cell–cell interactions and modulation of the immune response (Vimr *et al*., 2004; Varki, 2007).

The pathogens *Haemophilus influenza*,* Fusobacterium nucleatum*,* Pasteurella multocida* and *Vibrio cholera* have evolved complex and efficient methods to escape immune surveillance of the host by embellishing sialic acid as its surface antigen (Almagro‐Moreno and Boyd, 2009a,b). These organisms scavenge host‐derived sialic acid by importing it into their cytoplasm by a tripartite ATP‐independent periplasmic (TRAP) transporter (Severi *et al*., 2005; Allen *et al*., 2005). It can then be used either as a carbon and nitrogen source (catabolic pathway) or incorporated as a non‐reducing terminal sugar on the lipopolysaccharide (LPS) or lipooligosaccharide (LOS) (sialylation pathway) (Mulligan *et al*., 2010). As the sialic acid sialylation pathway enzymes of the bacteria are different from those in human beings in sequence and structure, they could be used as potential drug targets (Li and Chen, 2012).

**Figure 1:**
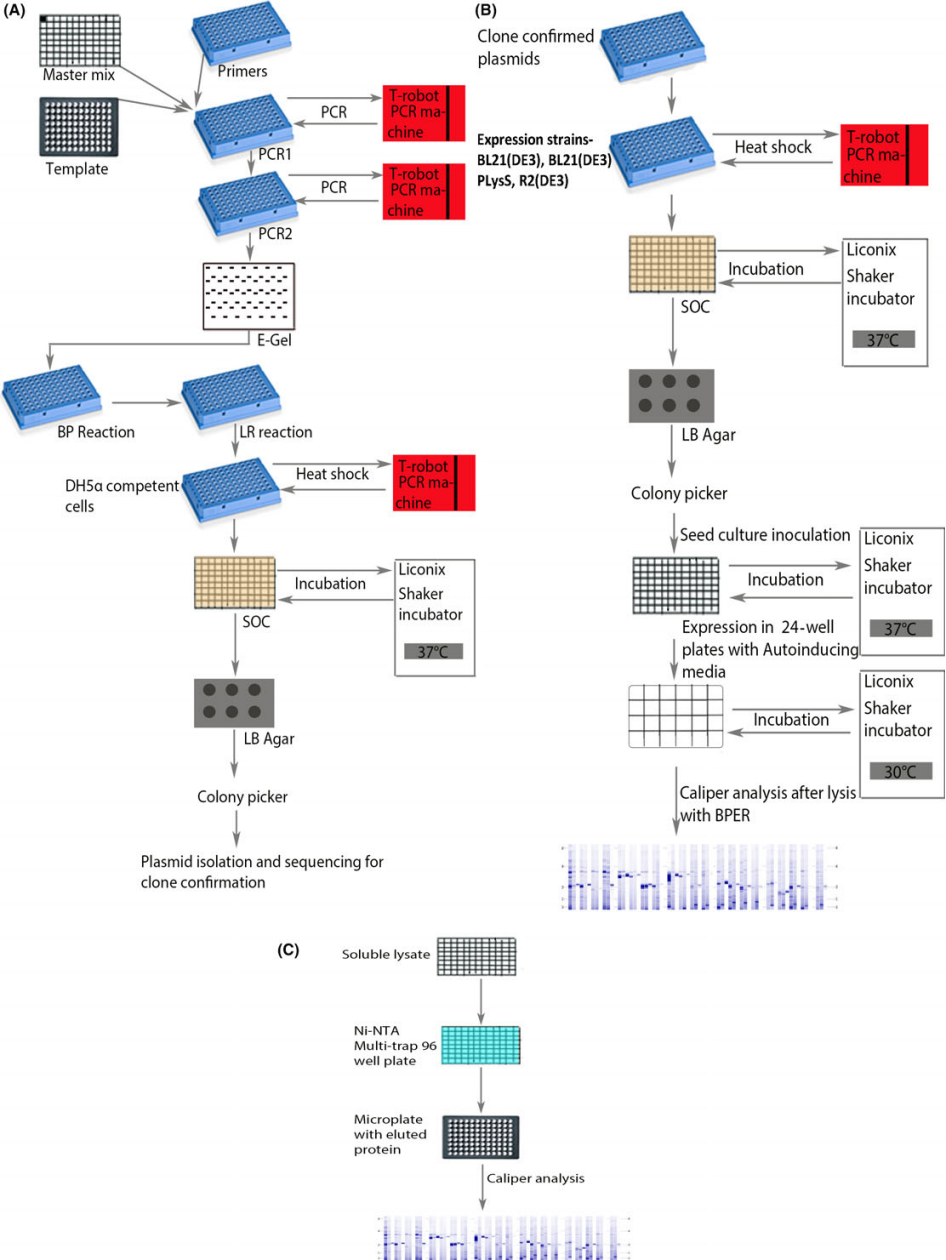
Flow charts for automating cloning, expression and purification on the liquid handling system. (A) Flow chart for automation of cloning. (B) Flow chart for automation of expression. (C) Flow chart for automation of purification. Primers, clone confirmed plasmids and competent cells are placed in PCR plates (blue plates); PCR and Gateway reactions (BP and LR reactions) are also carried out in PCR plates. Master mix, SOC and soluble lysates are provided in 96‐well deep‐well plates. LB agar is provided in 6‐well microplates (grey plate). Template and purified protein collected in 96‐well microplates (black plates). All the protocols were performed on the LHS (Liquid Handling System) which is integrated with 2 PCR machines, a spectrophotometer, an incubator with shaker, vacuum manifold, cooler rack, teleshake and E‐BASE (for running 96‐ and 48‐well DNA and protein gels). The user interface of the Freedom Evoware platform was used for writing and execution of all the programmes. All plate transfers were performed by RoMa (Robotic Manipulator) and liquid handling by LiHa (Liquid Handler).

Our main objective was to clone the genes involved in sialic acid catabolism and the surface sialylation pathway into a His‐tagged Gateway vector, followed by their small‐scale expression optimization *in vivo* to get the gene products in soluble form. *In vivo* expression was chosen over *in vitro* expression as the large‐scale studies were also to be performed *in vivo*. The constructs that showed best soluble expression were then selected for purification (Fig. 1) followed by scale up for crystallization trials. The further functional and structural characterization of these enzymes can be important for the development of new antimicrobial agents.

## Results and discussion

### Automation of PCR amplification followed by PCR clean‐up demonstrated for eighteen genes

Gateway cloning was used to streamline the cloning and expression process by alleviating costly, time‐consuming re‐cloning steps and avoiding the use of restriction enzymes in cloning and subcloning. All genes were synthesized by GeneArt in pMK vectors. Primers were designed for each of the genes to amplify the genes with 15 bp of the *att*B sites for cloning into the Gateway vector – pET300/NT‐DEST – containing an N‐terminal 6× His tag (first PCR amplification). A common primer pair for all the constructs was designed to add the remaining 15 bp of the *att*B sites to the PCR amplified products from the first PCR (second PCR amplification).

**Figure 2:**
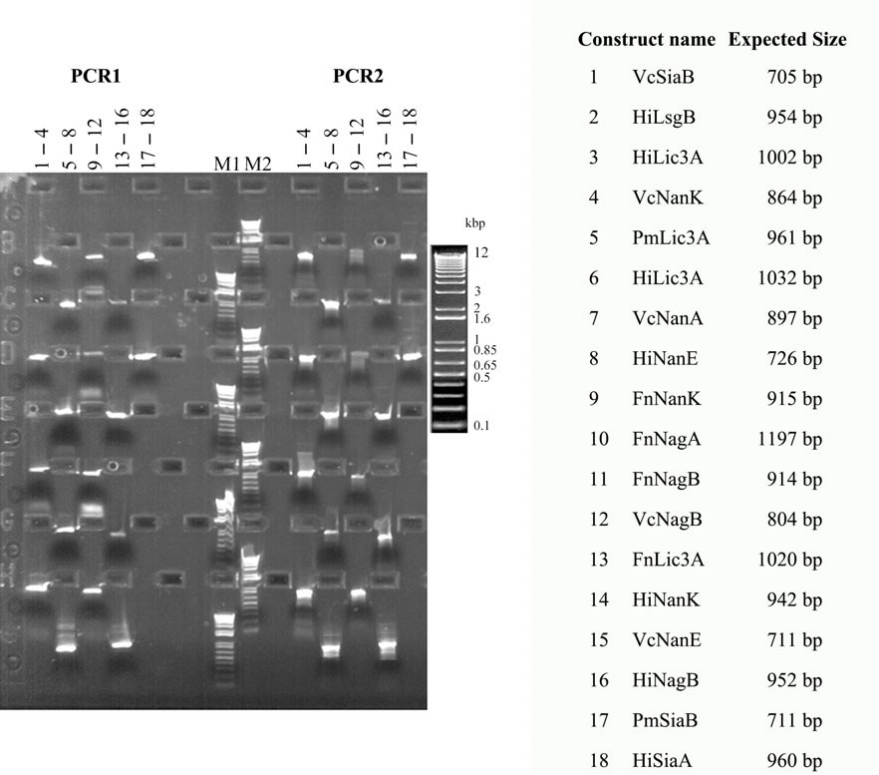
E‐PAGE images of PCR1 and PCR2 steps of cloning: Each vertical column of the E‐gel consists of four lanes one below the other (visible as rectangular boxes). PCR1 and PCR2 reactions of constructs 1–4 are run in columns 1 and 11 respectively. PCR1 and PCR2 reactions of constructs 5–8 are run in columns 2 and 12 respectively. PCR1 and PCR2 reactions of constructs 9–12 are run in columns 3 and 13 respectively. PCR1 and PCR2 reactions of constructs 13–16 are run in columns 4 and 14 respectively. PCR1 and PCR2 reactions of constructs 17–18 are run in columns 5 and 15 respectively. M1 and M2 are marker columns where marker is run in all four lanes for comparison with other lanes. The table on the right gives name and size details of the constructs. Phusion polymerase was used for all amplifications with an annealing temperature of 60°C, for 25 cycles. Phusion master mix was provided in a single well of a 96‐well deep‐well plates. Diluted primers and templates were provided in 96‐well PCR plates. All the components were mixed in a PCR plate on the cooler rack, and the PCR plate was transferred into the PCR machine on the LHS platform by RoMa (Robotic manipulator).

Amplification success rate was not satisfactory when the annealing temperature of the primers for the first PCR was low. Hence, longer primers were used with higher annealing temperatures of about 60°C for the first PCR (This set that gave successful results is listed in Table S2). All the constructs were amplified in the thermocycler with the same conditions resulting in PCR products with attB1 and attB2 sites on the 5′ and 3′ ends respectively (Fig. 2).

The PCR products were diluted three times with water and run on the 96‐well E‐gel. The amplified products were then cleaned up using Agencourt magnetic bead‐based PCR clean‐up kit and Qiagen filter‐based PCR clean‐up kit. The final elution was performed with 50 μl of Gibco Ultrapure water in a half‐area microplate. This allowed us to compare and understand the differences in the cloning efficiency from the purified products. The DNA concentration of the purified PCR products was then normalized to 70 ng μl^−1^ on the Tecan liquid handler. Picogreen was used along with DNA solutions with known concentrations to first quantify the DNA samples in a 384‐well plates using the spectrophotometer integrated to the LHS. The quantified values were used for normalization of PCR‐purified products. The normalization was performed using an existing normalization wizard in the freedom evoware software.

The normalized PCR products were then subjected to BP clonase and LR clonase recombination reactions according to the manufacturer's instructions (Invitrogen). The reactions were performed for 3 h each at room temperature. One μl of the LR recombination reaction was used for transformation into *E. coli* strain DH5α. We found that PCR products purified from the Agencourt Ampure beads gave lesser background colonies compared with the Qiagen PCR‐purified products. This was because of the ~100 bp primer dimers that were forming in the second PCR reaction. These dimers were not removed completely with the Qiagen PCR clean‐up kit, which has a cut‐off of around 70 bp; this results in the integration of these primer dimers into the gateway vector.

### Automated transformation and screening for positive clones

One μl of the LR recombination reaction was used for transforming the ultracompetent DH5α cells, 50 μl of which was prealiquoted in each well of a 96‐well PCR plates. After performing the heat‐shock protocol in the integrated PCR machine (45 s at 42°C followed by 2 min at 4°C), the cells were transferred to a 96‐well deep‐well plates containing super optimal broth with catabolite repression (SOC) followed by incubation for an hour at 37°C in the Liconix shaker incubator.

**Figure 3:**
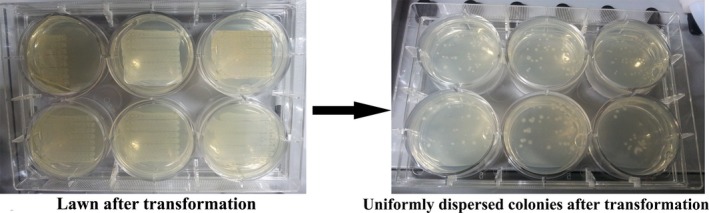
Optimization of transformation process: The plate on the left shows formation of lawn after transformation, before optimization. The plate on the right shows nicely dispersed colonies after optimization of transformation.

The plating of transformed cells was performed by two simple methods. In the first method, 0.5 μl of transformed culture was spotted 48 times using LiHA (Liquid Handling) tips evenly across the respective well in the six‐well plates. The second method was to directly dispense 100 μl ten times diluted culture in the centre of the plate, which eventually spreads across the plate when the plate is dried. The former method resulted in well‐dispersed colonies in the six‐well plates; however, it required 2.5 min to complete the plating of each sample. This translates to a very long time where higher numbers of samples are involved (Fig. 3). The latter method, however, is much quicker and also gave a decent dispersion of colonies. Hence, we applied diluted culture in the plate followed by drying for the subsequent plating steps involved in our experiments.

Three well‐defined colonies for each construct were then picked by the colony picker and inoculated into 30 μl of water. 5 μl was used for colony PCR with T7 primers, and the remaining 25 μl was used for inoculation into LB media for plasmid amplification. The plasmids were isolated on the sigma 96‐well filter‐based kit followed by sequencing using T7 forward and reverse primers.

### Expression

Once the sequence was confirmed, a clone for each construct was transformed into three different expression strains – BL21(DE3), BL21(DE3)PlysS (for tight regulation of expression; useful especially if the gene is slightly toxic) and Rosetta2 (DE3) (R2(DE3)). R2(DE3) consists of seven extra tRNAs, useful for genes containing rare codons (Novagen)). Normal chemical competent cells were used this time as opposed to ultracompetent cells, as we were transforming circular plasmids, not BP/LR cloning mixtures. Use of ultracompetent cells at this stage would result in a lawn, thus, making picking of single colonies difficult (Fig. 3). The obtained colonies were picked and inoculated into a 96‐well plates for seed culture using the colony picker. After overnight growth, 1% of the seed culture was inoculated into 1 ml of either 2X YT media or 1 ml of ZYM without lactose in a 96‐well deep‐well plates for expression in 2X YT or ZYM autoinduction media, respectively, in a 24‐well plates. A 24‐well plates were chosen here to enable the use of the higher volume of the culture. Several volumes – 2 ml, 3 ml and 4 ml volumes were tested for growth with one of the constructs at the maximum possible shaking speed of 1200 rpm, 1 mm orbital in the Liconix shaker. Sedimentation of cells was observed at volumes higher than 2 ml. Mixing and prevention of cell sedimentation were found to be best when 2 ml culture was used.

### Media optimization

IPTG‐induced expression was performed using 2X YT (containing tryptone and yeast extract) media. The seed culture (1%) from the 96‐well deep‐well plates was transferred into 2 ml media in 24‐well plates. After 3 h of growth (at 37°C) postinoculation, 0.4 mM IPTG was added along with lowering of the temperature to 30°C for 5 h. Fewer constructs expressed in 2X YT, possibly owing to the fact that different constructs reach their optimum induction optical densities (OD) at different times. Hence, generalizing conditions for all constructs in IPTG‐based expression may not be optimal. As an alternative, the autoinducing ZYM (Studier, 2005) media was used for expression at 30°C for 24 h. The slightly longer incubation times are to compensate the longer time required for the growth in the Liconix shaker incubator. It was seen that in autoinduction media unperturbed expression of all constructs occurred simultaneously.

**Figure 4:**
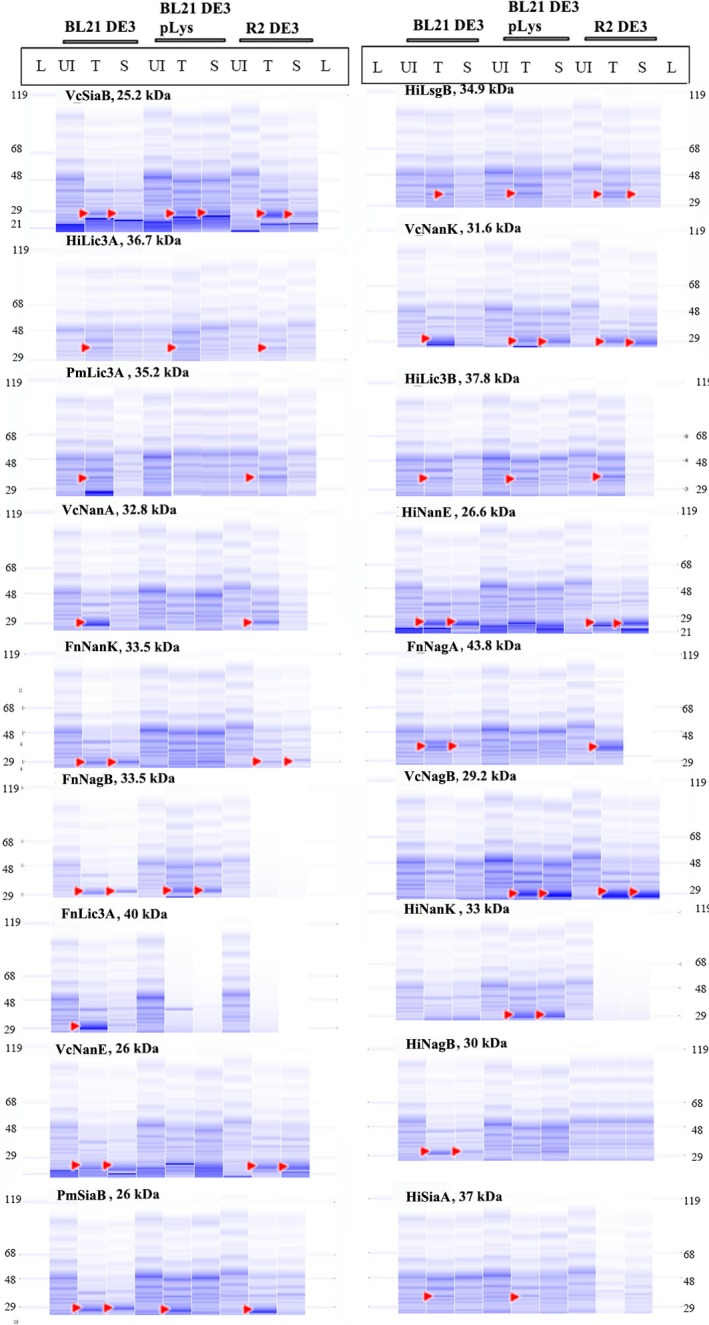
Analysis of expression of the 18 constructs using capillary electrophoresis on Caliper GXII system: L, Ladder lane; UI, Uninduced lysate; T, Total lysate; S, Soluble lysate. 10 μl of the total lysate sample was taken for analysis followed by a spin at 4000 rpm for 30 min to obtain the soluble fraction. 10 μl of the soluble fraction was taken for analysis, and the remaining supernatant was transferred to a fresh 96‐well deep‐well plates. 2 μl of the samples was required for analysing them on the Caliper instrument.

Expression and solubility were analysed by normalizing the cell pellet based on final OD (at 600 nm in a half‐area 96‐well microplates) and lysing the cells using Bacterial Protein Extraction Reagent (BPER). The sample with the lowest absorbance is re‐suspended in 200 μl of BPER, and the volume was adjusted according to the absorbance for all other samples by the normalization wizard. The samples were transferred into a 96‐well deep‐well plates and incubated at room temperature for 15 min. The total lysate and the soluble fractions were then run on Caliper GX. 12% E‐PAGE (polyacrylamide‐based SDS gel – compatible with liquid handling platform) was also used to run the samples. Even with the majority of samples running as a smear; we could easily observe overexpression; however, it was with difficulty that underexpression was observed. The normalized samples were also analysed on Labchip GX protein chip. Here, the resolution of separation allowed clear visualization of overexpressed and lesser expressed samples (Fig. 4).

Here, we found constructs mentioned in Table S3 to give soluble protein expression. These soluble proteins were further prepared in large scale for crystallization studies. In future, we intend to use a variety of solubility tags to improve the percentage of soluble constructs.

### Purification and scale up for crystallization studies

**Figure 5:**
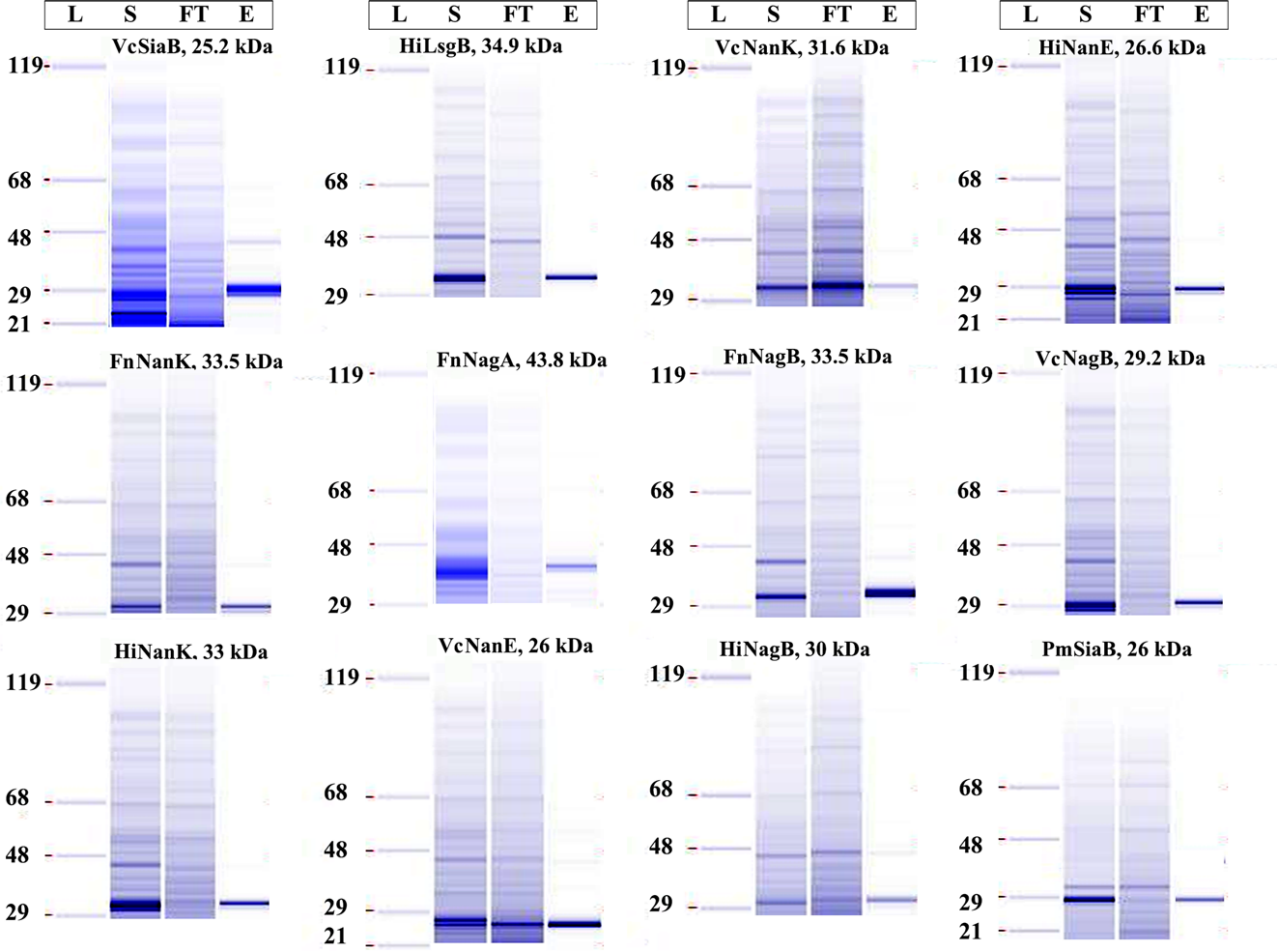
Analysis of purification of the soluble lysates on Caliper GXII system: L, Ladder; S, Soluble lysate; FT, Flow through; E, Elute. Using the vacuum manifold, the Ni‐NTA beads were first washed with water followed by equilibration with 500 μl of binding buffer (20 mM Tris (pH 7.5), 150 mM NaCl, 10 mM Imidazole). The lysate is then passed through followed by washing with binding buffer twice (500 μl each time). Bound protein is then eluted in 50 μl of elution buffer (20 mM Tris (pH 7.5), 150 mM NaCl, 500 mM Imidazole). The load, flow through and elutes are collected separately are then analysed on CaliperGX.

For each construct, the strain that showed best solubility was taken for further purification studies. All the constructs were His‐tagged and hence Nickel‐NTA‐based 96‐well filter plates were used for the purification process. The purified products were visualized by running on chip‐based Caliper GXII system. More than 95% purity was observed for most constructs (Fig. 5). Screening for soluble protein expression was much quicker through utilization of our automation platform.

**Figure 6:**
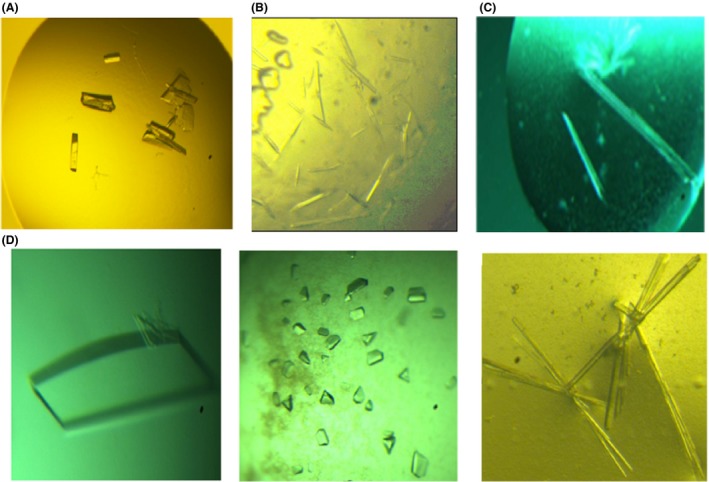
Crystal images of some of the proteins that were successfully crystallized. (A) Crystal images of FnNanA. (B) Crystal image of FnNanE. (C) Crystal images of VcSiaB. (D). Crystal images of PmNanK with ManNAc (Cocrystallization), PmNanK with AMP/PMP (soaking) and HiNanK with ManNAc and ATP (ADP) (Cocrystallization) from left to right.

After testing more constructs for solubility, four additional constructs (FnNanA, PmNanK, FnNanE and PmNagB) were included for large‐scale expression and crystallization. Table S3 lists the primers used for amplification of these constructs. Table S5 lists the expression and solubility conditions for the four constructs. The soluble proteins were expressed in large scale and were purified first by Ni‐NTA affinity chromatography followed by size exclusion chromatography to obtain high purity. The purified proteins were then set up for crystallization in trays by hanging drop diffusion method. Examples of crystal images are depicted in Fig. 6.

Our automation pipeline for cloning, expression and purification is demonstrated here for eighteen target proteins. Various strategies were attempted at each step, and the best‐suited strategy for automation was established at every stage. In several previous automation set‐ups for cloning, expression and purification, either only the initial few steps are automated or the steps are automated in different modules on different platforms. For example, Georg Mlynek *et al*. automated many of the steps in cloning, expression and purification, but with many manual interventions such as plating on agar after transformation, colony picking, and inoculation, analysis of DNA after PCR and protein using gel electrophoresis after expression and purification. Yehezkel *et al*. (2011) automated cloning until the step of transformation, whereas expression and purification steps were not automated. Here, we have developed automated cloning, expression and purification yielding in a fast and economic method with minimal manual intervention. The combination of the equipment and the processes used here for integrating and automating cloning, expression and purification has not been used before to the best of our knowledge. We intend to further expand the set‐up to include other applications such as media optimization, enzyme engineering through directed evolution. For example, enzyme engineering through directed evolution generally involves PCR amplification followed by cloning, transformation and screening. These activities are easily achievable on our platform with modifications in the existing programme.

## Author contributions

Conceived the project: MN, RF, SR. Conceived and designed the experiments: MN. Automation and integrations of all components designed: MN. HTP experiments performed by: MN, SGB, Large‐scale expression, and crystallization performed by RCC, RF, TGS, SS, LM, SB, JPK, SRG and VN. Analysed the data: MN, SGB, LNG. Contributed reagents/materials/analysis tools: VN, MN, SR. Wrote the paper: MN, SGB, RF, SR while all authors discussed the results and made manuscript revisions.

## Conflict of interest

None declared.

## Supporting information


**Table S1.** Genes cloned along with accession numbers.
**Table S2.** Primer list for first PCR amplification of 18 constructs
**Table S3.** Solubility of the 18 constructs.
**Table S4.** Primer list for first PCR to amplify FnNanA, PmNanK, FnNanE, PmNagB.
**Table S5.** Solubility of FnNanA, PmNanK, FnNanE and PmNagB.
**Appendix S1.** Supplementary methods.
