# Supplementary material for: Automation aided optimization of cloning, expression and purification of enzymes of the bacterial sialic acid catabolic and sialylation pathways enzymes for structural studies
Source: Microb Biotechnol. 2018 Jan 17;11(2):420–8. doi: 10.1111/1751-7915.13041 (PMC5812244; doi:10.1111/1751-7915.13041)
Supplement: Supplementary file 1 — Table S1. Genes cloned along with accession numbers. Table S2. Primer list for first PCR amplification of 18 constructs Table S3. Solubility of the 18 constructs. Table S4. Primer list for first PCR to amplify FnNanA, PmNanK, FnNanE, PmNagB. Table S5. Solubility of FnNanA, PmNanK, FnNanE and PmNagB. Appendix S1. Supplementary methods. [file MBT2-11-420-s001.docx]

**Supplementary Information**

**Supplementary Table 1: Genes cloned along with their accession numbers**

| **Gene name** | **Gene accession number** | **Organism** |
| --- | --- | --- |
| VcNanA | NP_231411.1 | *Vibrio cholerae* O1 biovar E1 Tor str. N16961 |
| VcNanK | NP_231417.1 | *Vibrio cholerae* O1 biovar E1 Tor str. N16961 |
| VcNanE | NP_231416.1 | *Vibrio cholerae* O1 biovar E1 Tor str. N16961 |
| VcNagB | WP_001237050 | *Vibrio cholerae* O1 biovar El Tor str. N16961 |
| VcSiaB | WP_000064388 | *Vibrio cholera* |
| HiSiaA | P44066 | *Haemophilus influenzae* KW20/RD |
| HiLic3B | ABR14150 | *Haemophilus influenzae* |
| HiLsgB | Q4QJP2 | *Haemophilus influenzae* 86-028NP |
| HiNagB | Q4QP46 | *Haemophilus influenzae* 86-028 NP |
| HiNanK | Q4QP43 | *Haemophilus influenzae* 86-028NP |
| HiNanE | Q4QP42 | *Haemophilus influenzae* 86-028 NP |
| HiLic3A | Q4QNI8 | *Haemophilus influenzae* 86-028 NP |
| PmLic3A | V4NA83 | *Pasteurella multocida* subsp. *multocida* |
| PmSiaB | V4MRS1 | *Pasteurella multocida* subsp. *multocida* |
| FnNanK | Q8RDN7 | *Fusobacterium nucleatum* str ATCC 25586 |
| FnNagA | U7SKP0 | *Fusobacterium nucleatum* CTI-5 |
| FnNagB | R9RC00 | *Fusobacterium nucleatum* ssp. *animalis* 4_8 |
| FnLic3A | WP_005897393 | *Fusobacterium nucleatum* |

**Supplementary Table 2: Primer list for first PCR amplification of 18 constructs**

**Primer name Primer sequence**

VcSiaB_fp CAAAAAAGCAGGCTTC ATGTCGAATGAATATGTTGC

VcSiaB_rp CAA GAA AGC TGG GTT TCATTTATTAATTTCCTTAATTTTC

HiLsgB_fp C AAA AAA GCA GGC TTC ATGAATCTCATTCTTTGTT

HiLsgB_rp CAA GAA AGC TGG GTT TTAAATTTCCTTATAAATCTTTAA

HiLic3A_fp C AAA AAA GCA GGC TTC ATGTCAATCAATCAATCAATCA

HiLic3A_rp CAA GAA AGC TGG GTT CTAATCCCATTTTCTTGATTTTAA

VcNanK_fp CAAAAAAGCAGGCTTC ATGCGCACACTAGCCATC

VcNanK_rp CAA GAA AGC TGG GTT TTACTCCTTAAACTGAAATGCTG

PmLic3A_fp C AAA AAA GCA GGC TTC ATGGATAAGTTCGCAGAA

PmLic3A_rp CAA GAA AGC TGG GTT CTATTTTTCTTTTAAATAGTGTTTA

HiLic3B_fp C AAA AAA GCA GGC TTC ATGAACGGTACAATATGCCC

HiLic3B_rp CAA GAA AGC TGG GTT TTATTTGCGTAGTCTCATTTTCTT

VcNanA_fp CAAAAAAGCAGGCTTC ATGAAAAAACTAACAGGTTTG

VcNanA_rp CAA GAA AGC TGG GTT TTAGATCGACAGGAACCC

HiNanE_fp CAAAAAAGCAGGCTTCATGTCTAAATTATCTTATCAAGAAGTAC

HiNanE_rp CAAGAAAGCTGGGTTCTATCTTGCAGATTTCACTGAATTAG

FnNanK_fp C AAA AAA GCA GGC TTC ATGAATATTTTAGCAATAGAT

FnNanK_rp CAA GAA AGC TGG GTT TTATCTTTTATTAATTTTCTCT

FnNagA_fp C AAA AAA GCA GGC TTC ATGAAAAAAATATTATTAAAAAATG

FnNagA_rp CAA GAA AGC TGG GTT CTATCTTTTAAATTTGATTTTTC

FnNagB_fp C AAA AAA GCA GGC TTC ATGAGATTTATTGTAACTG

FnNagB_rp CAA GAA AGC TGG GTT TTATTTTTTATATAAGTTTTCAATT

VcNagB_fp CAAAAAAGCAGGCTTC ATGAGACTTATCCCACTGAAAG

VcNagB_rp CAA GAA AGC TGG GTT TTAGAAGCCTACGATGTTTTTG

FnLic3A_fp C AAA AAA GCA GGC TTC ATGAATTTATATATAATATATAACT

FnLic3A_rP CAA GAA AGC TGG GTT TTATTTTTCTATAAACTTATTTC

HiNanK_fp C AAA AAA GCA GGC TTCATGCGTTGTTTAGCACTAG

HiNanK_rp CAA GAA AGC TGG GTTTTAGCCATAAATTGTTCC

VcNanE_fp CAAAAAAGCAGGCTTC ATGAGAAAGAATTTTTTGAATATC

VcNanE_rp CAA GAA AGC TGG GTT CTAGTGTGCGCATTTCC

HiNagB_fp C AAA AAA GCA GGC TTCATGCGTTTTATTCCATTAC

HiNagB_rp CAA GAA AGC TGG GTTCTATTTATCCTGATAATCC

PmSiaB_fp C AAA AAA GCA GGC TTC ATGACAAATATTGCGATCATT

PmSiaB_rp CAA GAA AGC TGG GTTT TCATTTATTGGATAAAATTTCCG

HiSiaA_fp C AAA AAA GCA GGC TTCATGAAATTTGTTTCTATAATTAG

HiSiaA_rp CAA GAA AGC TGG GTTGGAAATATTGAGTATGAATAG

**Supplementary Table 3: Solubility of the 18 constructs**

**Constructs Expression in strains: Soluble in strains:**

VcSiaB BL21(DE3), BL21(DE3) pLys, R2(DE3) BL21(DE3), BL21(DE3) pLys, **R2(DE3)**

HiLsgB BL21(DE3), BL21(DE3) pLys, R2(DE3) None

HiLic3A BL21(DE3), BL21(DE3) pLys, R2(DE3) None

VcNanK BL21(DE3), BL21(DE3) pLys, R2(DE3) BL21(DE3) pLys, **R2(DE3)**

PmLic3A BL21(DE3), R2(DE3) None

HiLic3B BL21(DE3), BL21(DE3) pLys, R2(DE3) None

VcNanA BL21(DE3), R2(DE3) None

HiNanE BL21(DE3) **BL21(DE3)**

FnNanK BL21(DE3), R2(DE3) **BL21(DE3),** R2(DE3)

FnNagA BL21(DE3), R2(DE3) **BL21(DE3)**

FnNagB BL21(DE3), BL21(DE3) pLys BL21(DE3), **BL21(DE3) pLys**

VcNagB BL21(DE3) pLys, R2(DE3) BL21(DE3) pLys, **R2(DE3)**

FnLic3A BL21(DE3) None

HiNanK BL21(DE3) pLys **BL21(DE3) pLys**

VcNanE BL21(DE3), R2(DE3) BL21(DE3), **R2(DE3)**

HiNagB BL21(DE3) **BL21(DE3)**

PmSiaB BL21(DE3), R2(DE3) **BL21(DE3)**

HiSiaA BL21(DE3), BL21(DE3) pLys, R2(DE3) None

Strains in bold lettering indicate best solubility in comparison to the other strains

**Supplementary Table 4: Primer list for first PCR to amplify FnNanA, PmNanK, FnNanE, PmNagB**

| **Primer name** | **Primer sequence** |
| --- | --- |
| FnNanA_fp | C AAA AAA GCA GGC TTC ATGAAAGGGATATATTCAG |
| FnNanA_rp | CAA GAA AGC TGG GTT TTAATTTTTTAAAAATTTTTTATG |
| PmNanK_fp | C AAA AAA GCA GGC TTC ATGCGCTGTTTAGCATTAG |
| PmNanK_rp | CAA GAA AGC TGG GTT CTATTTCAAATGAACGCCTT |
| FnNanE_fp | CAAAAAAGCAGGCTTCATGAATAAAATTTTAGAAAGTATAAGAGG |
| FnNanE_rp | CAAGAAAGCTGGGTTTCATTTCATCTACAAACTTTTTAGTTATTTG |
| PmNagB_fp | C AAA AAA GCA GGC TTC ATGCGTTTAATTCCATTACACA |
| PmNagB_rp | CAA GAA AGC TGG GTT TTATTTCACGCTACGAATAGC |

**Supplementary Table 5: Solubility of FnNanA, PmNanK, FnNanE and PmNagB**

| **Constructs** | **Expression in strains:** | **Soluble in strains:** |
| --- | --- | --- |
| FnNanA | BL21(DE3), BL21(DE3) pLys, R2( DE3) | **BL21(DE3),** BL21(DE3) pLys, R2 (DE3) |
| PmNanK | BL21(DE3) pLys | BL21(DE3) pLys |
| FnNanE | BL21(DE3), BL21(DE3) pLys, R2 (DE3) | **BL21(DE3),** BL21(DE3) pLys, R2 (DE3) |
| PmNagB | BL21(DE3), BL21(DE3) pLys, R2 (DE3) | **BL21(DE3),** BL21(DE3) pLys, R2 (DE3) |

Strains in bold lettering indicate best solubility in comparison to the other strains

**Supplementary methods**

**Crystallization conditions:**

Crystallization trials were carried out using standard hanging drop vapour diffusion techniques. Crystallization plates were set up using Mosquito nanoliter robot liquid handler from TTP labtech.

Conditions for nine of the proteins that were successfully crystallized are described below: **FnNanA** (N-acetylneuraminate lyase from *Fusobacterium nucleatum*): Purified protein was concentrated to 10mg/ml in 50mM HEPES pH 6.8, 50mM NaCl and 10mM β-mercaptoethanol for crystallization trials. The optimized crystals (diffraction to 2.3 Å resolution) were obtained in 10% (w/v) PEG-3000 and 0.1M CHES pH 9.5 at 4°C.

**PmNanK** (N-acetyl-mannosamine kinase from *Pasturella multocida*): Purified PmNanK was co-crystallized along with a 10-fold molar excess of ManNAc. Trays were set up for crystallization by hanging drop diffusion method. Drops were set up with equal volumes of protein and 0.2 M Sodium citrate tribasic dihydrate, 20% w/v PEG 3,350 and these were suspended over 100µl of crystallization buffer. These crystals diffracted to 2.7Å and they belong to space group C222_1_. PmNanK crystals with AMP-PNP were obtained by crystallizing the protein in 0.1 M Tacsimate pH 5.0, 20% (v/v) Isopropanol, 20%(w/v) PEG 4000, 20% v/v ethylene glycol. Later these crystals were soaked in AMP-PNP for 7 days to get crystals of PmNanK with the nucleotide. These crystals diffracted to 2.0 Å and belonged to the space group *P* 3_2_21.

**HiNanK** (N-acetyl-mannosamine kinase from *Haemophilus influenzae*): Purified HiNanK was co-crystallized with ManNAc and ATP by adding ManNAc and ATP to the protein at 1:10:10 concentration ratio. Trays were set up for crystallization by hanging drop diffusion method. Drops were set up with equal volumes of protein mix and 0.2M Ammonium phosphate monobasic, 0.1M Tris pH 8.5 and 50% (v/v) (+/-)-2-Methyl-2,4-pentanediol. These crystals diffracted to 2.6 Å resolution and belong to space group I4_1_22.

**FnNanE** (N-acetylmannosamine-6-phosphate 2-epimerase from *Fusobacterium nucleatum*): Crystallization of the protein was carried out by the vapour diffusion method. Crystals were obtained when equal volumes of 18 mg/mL of protein were mixed with 0.4M MgCl2, 22% PEG 3350, 0.1M MES buffer pH5.2 and incubated at 18°C.

Purified **VcNanE** (N-acetylmannosamine-6-phosphtate 2 epimerase from *Vibrio cholera*) was concentrated to 28 mg/ml and crystallized in 20% PEG3350, 0.3M Malonate, pH 5.0.

**FnNagB**, **PmNagB** and **HiNagB** (Glucosamine 6-phosphate deaminase from *Fusobacterium nucleatum*, *Pasturella multocida* and *Heamophilus influenzae):* HiNagB crystals used for data collection (to 2.6 Å resolution) were obtained with hanging drop, vapour diffusion technique at 18°C in 2 % w/v Tascminate pH 5.0, 0.1 M Sodium Citrate tribasic pH 5.6, 16 % PEG 3350 containing 0.1 M Strontium Chloride as additive. The concentration of protein used was 15 mg/ mL. PmNagB crystals used for data collection (to 2.4 Å resolution) were obtained with hanging drop, vapour diffusion technique at 18°C in 100 mM Sodium Cacodylate/HCl pH 6.5, 200 mM Magnesium Chloride, 20 % PEG 1000. The concentration of the protein was 16 mg/mL. FnNagB crystals used for data collection (to 2.8 Å resolution) were obtained with hanging-drop, vapour diffusion technique at 18°C in 100 mM HEPES/NaOH pH 7.5, 200 mM NaCl, 30 % PEG 400, containing 0.1 M Manganese (II) chloride as additive. The concentration of the protein was 21 mg/mL.

**VcSiaB** (CMP-Sialic acid Synthetase from *Vibrio cholerea*): Purified VcSiaB was crystallized using hanging drop vapour diffusion at 18°C. 0.5 µl aliquot of the VcSiaB protein (10 mg/ml) was mixed with 0.5µl of 200mM calcium acetate, 0.1M imidazole pH 8.0 and 10% PEG8000 to obtain crystals. Rod shaped crystals appeared within 3 days and continued to grow for a week. The crystals were cryo-protected and data was collected in SOLEIL-PROXIMA I beamline. The crystals diffracted to 2.6 Å resolution and processed to P4_1_ spacegroup.
